# Vitamin D deficiency among healthy adolescents in Al Ain, United Arab Emirates

**DOI:** 10.1186/1471-2458-13-33

**Published:** 2013-01-14

**Authors:** Shamma J Muhairi, Aaesha E Mehairi, Aysha A Khouri, Muna M Naqbi, Fatima A Maskari, Juma Al Kaabi, Ayesha S Al Dhaheri, Nico Nagelkerke, Syed M Shah

**Affiliations:** 1 United Arab Emirates University, College of Medicine and Health Sciences, Department of Family Medicine, Al Ain, UAE; 2 United Arab Emirates University, College of Medicine and Health Sciences, Institute of Public Health, PO Box 17666, Al Ain, UAE; 3 United Arab Emirates University, College of Food & Agriculture, Department of Nutrition and Health, Al Ain, UAE; 4 United Arab Emirates University, College of Medicine and Health, Department of Internal Medicine, Al Ain, UAE

**Keywords:** Vitamin D deficiency, Adolescents, United Arab Emirates

## Abstract

**Background:**

Although vitamin D deficiency has been studied in various adult populations, there are few data on the prevalence of this nutritional deficiency among healthy adolescents in the United Arab Emirates (UAE). This study was conducted to determine the prevalence of vitamin D deficiency and to examine its correlates in adolescents aged 15 to 18 years.

**Methods:**

This was a cross-sectional study in urban schools. Healthy adolescents (N=315) from a sample of 8 schools were randomly selected from the 142 schools in Al Ain, Abu Dhabi Emirate. Outcomes measured included serum concentrations of 25-hydroxy vitamin D (25OHD), plasma lipids, blood sugar, blood pressure and anthropometric data, nutrition and lifestyle variables.

**Results:**

Fourty-one participants (19.7%) were vitamin D deficient (serum 25OHD level ≤15 ng/mL [≤37.5 nmol/L]. Using a cutoff level of 25(OH) D of ≤20 ng/ml [≤50 nmol/l] 143 participants (45.4%) were vitamin D insufficient. Overall 65.1% of study participants were either vitamin D deficient or insufficient. The prevalence of vitamin D deficiency varied between boys (10%) and girls (28%). In a final multivariate model, serum 25(OH) D concentrations were inversely correlated with female gender, consumption of fast food per week, and body mass index and positively correlated with physical activity scores after adjustment for age.

**Conclusions:**

Vitamin D deficiency and insufficiency were highly prevalent in adolescents, and more common in girls.

## Background

Inadequate exposure to sunlight and low nutritional intake of vitamin D result in low serum concentrations of circulating 25(OH) D, a condition known as hypovitaminosis D [1,2]. Severe vitamin D deficiency in children leads to nutritional rickets and it is associated with developmental delays and impaired growth [3]. Therefore, adequate stores of vitamin D are crucial for musculoskeletal health, especially since peak bone mass achieved early in life is a predictor of osteoporosis risk in adulthood [4]. In addition, there is a growing body of evidence that vitamin D deficiency is associated with type 1 diabetes [5] and cardiovascular disease (CVD) risk factors such as hypertension, hyperglycemia and metabolic syndrome [6-8]. The extreme consequences of vitamin D deficiency such as rickets in children and osteomalacia in adults have been almost eliminated in some developed countries through adequate diet, food fortification, and the encouragement of moderate sunlight exposure [9].

Vitamin D deficiency remains a problem globally [10,11]. Vitamin D deficiency in women in Arab countries has been attributed to inadequate exposure of skin to sunlight due to a very conservative style of dress (e.g. niqab, hijab) that covers most of the body when they are outside [12,13]. In a recent study, Arab-American women practicing a conservative style of dress showed a significantly higher prevalence of vitamin D deficiency compared to their counterparts practicing a less conservative style of dress [14]. In the United Arab Emirates (UAE), female youth similarly maintain a very conservative style of dress that limits sunlight exposure. Population-based data are lacking about the status of vitamin D in UAE adolescents. Therefore, the objectives of this study were to determine the prevalence of 25-hydroxyvitamin D (25[OH] D) deficiency and its correlation with gender, diet and CVD risk factors.

## Methods

A cross-sectional study of healthy adolescents aged 12 to 18 years was conducted in Al Ain city in the United Arab Emirates during March and April of 2010. Abu Dhabi is the largest of the seven emirates that make up the United Arab Emirates and the city of Al Ain (“the spring” in Arabic) is a fertile oasis city located approximately 160 kilometers east of the Abu Dhabi capital.

Study participants were recruited as part of a metabolic syndrome project where 8 schools out of a total of 142 schools were randomly selected and adolescents in grades between 6 and 12 enrolled. The targeted sample size (n=1630) was selected with probability proportional to the school size. At the second stage, classes in each school were randomly selected by using the even and odd numbers of each grade. The final sample (n=1018) included participants who had fasted for at least 8 hours, agreed to draw blood, and had not used any regular medications or had any chronic medical conditions that might affect growth, body composition, dietary intake or physical activity and cigarette smoking. Serum 25(OH) D concentrations were measured in a sub-sample of participants, aged 15 to 18 years (n=315). The latitude of schools did not change in Al Ain city. We did not collect information on the use of sunscreen.

The study protocol was approved by the Human Research Ethics Committee of the Al Ain, Medical District. All the participants and their parents provided informed consent.

### Data collection

Personal identification numbers were assigned to each participant to maintain anonymity. The identification number was used to confirm consent status and to link students to their respective schools, clinical measurements and blood samples. A questionnaire was developed to obtain relevant information related to age, gender, ethnicity, mother’s education level, dietary habits, and physical activity. The questionnaire was translated in Arabic and validated by a pilot test using 30 volunteers. In the dietary habit section of the questionnaire, milk and milk product consumption data were categorized as less than once per day, once per day and more than once per day. The short version of the International Physical Activity Questionnaire [14] was used to assess the physical activity status in the study participants. Part of the physical activity questionnaire asked information on hours spent watching television, playing video games, and computer use. Combined television, video, and computer hours were categorized as none, ≤2 hours, 3 to 4 hours and >4 hours per day.

### Measurements and laboratory analysis

A training workshop on standardizing the method of anthropometric and blood pressure measurement was conducted by qualified trainers prior to data collection, and involved nurses of all study schools. All measurements were performed at the same time of the day (i.e. early morning between 8 and 11 am) for all participants. Height was measured in centimeters (cm) using a stadiometer (SECA) with the participant standing in an upright position without wearing shoes. Waist circumference was measured in centimeters (cm) using an un-stretched measuring tape placed around the midpoint between the bottom of the rib cage and above the tip of the iliac crest. Body weight was measured to the nearest 0.2 kilograms (kg) using a digital scale (SECA) with the participant standing in an upright position without shoes and in light clothing. Triplicate readings of height, weight and waist circumference were taken and the average was considered to be the participant’s measurement.

Following at least five minutes of rest in a seated position, blood pressure (BP) was measured on the right arm using a standard mercury sphygmomanometer with an appropriate cuff size. All participants were required to refrain from smoking, consuming caffeine and participating in any moderate- or vigorous-intensity physical activity at least 60 minutes before their blood pressure measurement. Three consecutive measures were obtained at one-minute intervals and the average of the last two readings was considered to be the participant's blood pressure.

A five milliliter venous blood sample was obtained from the subjects by qualified nurses using standardized tubes. Prior to blood sampling, all participants had been instructed to fast for at least eight hours and abstain from smoking. Blood samples were sent to Tawam hospital laboratory within two hours of blood draw where they underwent standardized (quality controlled) analyses. Serum 25 (OH) D concentrations were measured by radioimmunoassay (DiaSorin, Stillwater, MN). The intra-assay and inter-assay for coefficients of variation were 8.3% and 3.2% respectively. The fasting plasma glucose (FBS), high density lipoproteins (HDL) and triglycerides (TG) were analyzed by the DXC 800 Analyzer (Beckman Coulter, Fullerton, CA, USA) using the appropriate conventional laboratory reagents, enzymatic and calorimetric techniques.

### Definitions

Body mass index (BMI) was calculated as body weight in kilograms divided by height in meters squared. BMI was used to classify participants as either healthy/normal weight (BMI 5^th^ to 75^th^ percentile), overweight (BMI between the 85^th^ and 95^th^ percentiles) or obese (BMI ≥95^th^ percentile) according to the 2000 Centers for Disease Control and Prevention growth charts using Anthro program in Epi-Info software [15].

Metabolic syndrome (MetS) was defined using the diagnostic criteria proposed by the International Diabetes Federation [16]; namely, waist circumference ≥90^th^ percentile or ≥94^th^ percentile (for youth aged 16 years or older), triglycerides concentrations ≥150 mg/dL(1.7 mmol/L), HD-cholesterol <40 mg/dL(1.03 mmol/L) or <50 mg/dL (1.29 mmol/L) for female adolescents aged ≥16 years and older, fasting plasma glucose (FPG) concentrations of >100mg/dL (5.6 mmol/L) and blood pressure (BP) ≥130/80 mmHg.

Elevated blood pressure was defined by using percentiles for systolic and diastolic values on the basis of height percentile, age and gender. Values >95th percentiles were considered elevated [17].

The 25 (OH) levels are the most commonly measured indicator of vitamin D status. We defined vitamin D deficient as having serum 25OHD level ≤15 ng/mL [≤37.5 nmol/L] and vitamin D insufficient as 25OHD level ≤20 ng/mL [≤50 nmol/L], respectively [18,19].

### Statistical analysis

All data were normally distributed. Descriptive statistics (frequencies, means, standard deviations) were used to estimate serum 25(OH) D concentrations by age group, gender, nationality, mother’s education level, food intake, physical activity, nutritional status and by presence of cardiovascular risk factors such as high blood pressure, high blood glucose, low HDL-cholesterol, high triglycerides and metabolic syndrome. The analyses were conducted using Epi-info, and SPSS (v.19). A one way ANOVA and student t-test was used to analyze the association between serum 25(OH) D concentrations and potentially associated variables. Statistical significance was defined as p-values <0.05. Serum 25 (OH) D concentrations were considered as a continuous outcome variable for multivariate analyses to avoid power loss associated with categorization [20] and stepwise linear regression analysis was used to identify the significant covariates of serum 25 (OH) D concentrations.

## Results

The age of study participants (n=315) ranged from 15 to 18 years and 52% were female (n=165). The mean age (SD) was 16 years for females (0.6) as well as for males (0.9). The mean distribution of 25(OH) D concentrations across characteristics of 315 study participants is listed in Table 1. There were no significant differences in 25(OH) D concentrations between Emirati youth and youth from other Gulf and Arab countries. The majority of the study participants (63%) were Emirati nationals and the rest (37%) were from other Arab and Gulf countries.

**Table 1:** Mean 25 (OH) D concentrations by socio-demographic characteristics, physical activity, and dietary habits of UAE adolescents aged 12 to 18 Years, 2010

**Characteristic**	**All**	**25 (OH) D Level, ng/mL**	
	**n**	**Mean (95%CI)**	**p-value**
Overall	315	23.8 (22.1-24.7)	NA
**Age, Y**			
15-16	142	24.2 (22.4-25.9)	
17-18	173	23.1 (21.8-24.6)	0.373
**Gender, %**			
Male	150	26.2 (24.6-27.8)	
Female	165	21.2 (19.8-22.7))	0.000
**Nationality**			
Emirati	198	23.2 (21.8-24.3)	
Other Gulf or Arab Countries (References)	117	24.6 (21.3-26.5)	0.364
**Mother education**			
No formal education	47	21.5 (19.2-23.8)	
Up to secondary	162	23.5 (22.0-25.0)	
College/University	49	24.8 (21.3-28.2)	0.127
**Physical Activity Score**			
Low	115	22.1 (20.2-23.9)	
Moderate	102	23.0 (21.2-24.8)	
High	95	26.3 (24.3-28.3)	0.005
**Television and Computer Use, h/day**			
Non	27	27.1 (22.5-31.7)	
Up to 2 hours	99	24.6 (22.7-26.5)	
More than 2 hours	186	22.5 (21.3-23.9)	0.030
**Milk and milk product intake**			
Less than once per day	141	22.3 (21.0-23.7)	
Once per day	85	24.8 (22.3-27.3)	
More than once per day	89	24.4 (22.3-26.5)	0.127
**Meals at Fast Food in past 7 days**			
Never	64	26.2 (23.3-29.1)	
Once per week	148	23.4 (21.8-24.9)	
More than once per week	103	22.3 (20.6-24.1)	0.042
**Carbonated soft drink intake in a day**			
Less than once a day	171	23.8 (22.3-25.3)	
Once or two times a day	70	21.4 (19.6-23.1)	
More than two times a day	74	25.2 (22.6-27.8)	0.058
**Vegtables intake in a day**			
Less thance once a day	170	23.1 (21.6-24.4)	
Once daily	71	23.8 (21.1-26.4)	
More than once a day	74	24.8 (22.5-27.0)	0.417

**Table 2:** Mean 25 (OH) D levels by cardiovascular disease risk factors of UAE adolescents aged 12 to 18 years (n=315), AL Ain, 2010

**Variable**	**All**	**Mean 25 (OH) D Level, ng/mL**	
	**n**	**(95% CI)**	**p-value**
BMI			
<85 percentile	212	23.8 (21.9-25.9)	
≥85th percentile	50	25.8 (22.5-29.2)	
≥95th percentile	52	20.6 (18.2-22.4)	0.023
Waist circumference ≥90th percentile			
Yes	25	20.5 (17.8-23.2)	
No	290	23.9 (22.7-25.0)	0.101
High blood pressure			
Yes	35	23.2 (20.5-25.9)	
No	279	23.7 (22.5-24.9)	0.342
Fasting Glucose ≥100 mg.dL			
Yes	17	21.3 (18.5-24.1)	
No	298	23.7 (22.6-24.9)	0.098
HDL-cholesterol ≤40 mg mg/dL			
Yes	174	24.9 (23.4-26.4)	
No	141	22.1 (20.5-23.6)	0.011
Triglycerides ≥ 110 mg/dL			
Yes	16	23.2 (17.8-28.5)	
No	299	23.6 (22.5-24.8)	0.860
Metabolic syndrome			
Yes	26	22.0 (19.7-24.2)	
No	288	23.8 (22.6-24.9)	0.151

Out of 315 study participants, 41 (19.7%) were vitamin D deficient (serum 25OHD concentrations ≤15 ng/mL [≤37.5 nmol/L]. Using a cutoff of (25OHD concentrations ≤20 ng/mL [≤50 nmol/L], 143 participants (45.4%) were vitamin D insufficient. We removed 6 participants who were on medication for diabetes or hypertension. Only boys reported (15%) current cigarette smoking and none of the girls had history of current smoking.

**Figure 1:**
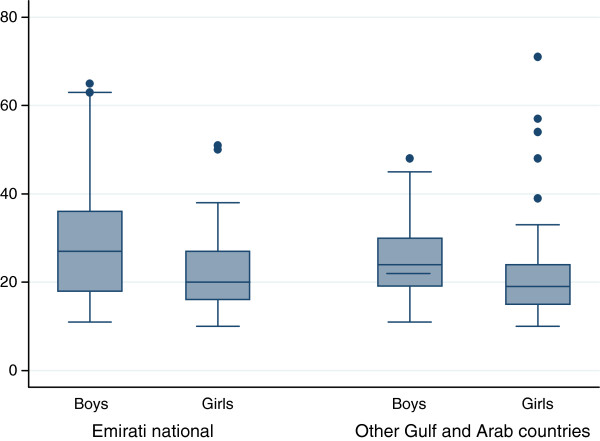
Box plot of participants’ serum 25-hydroxyvitamin D (25OHD) concentration (ng/mL) by gender and nationality, (n=315), Al Ain, United Arab Emirates, 2010.

Among Emirati adolescents, a higher proportion of females (32.0%) were vitamin D deficient compared to their male counterparts (8.0%) and this difference was significant (p<0.05). Similarly female adolescents from other Gulf and Arab countries had higher prevalence of vitamin D deficiency (23.5%) compared to their male counterparts (14.3%) and this difference was significant (p<0.05). Mean serum 25 (OH) D concentrations was 20.8 ng/mL (95% CI 18.8-22.8) in Emirati females as compared to Emirati males (mean 25.2 ng/mL; 95% CI 23.7-26.8). Mean serum 25 (OH) D concentrations was 21.9 ng/mL (95% CI; 19.8-23.9) in female adolescents from other Gulf and Arab countries compared to their males counterparts (mean 28.3; 95% CI 24.6-31.9) as shown in Figure 1.

Mean 25 (OH) D concentrations were highest in those who had the highest physical activity score, intermediate among adolescents with moderate physical activity and the lowest in those with low physical activity score (p<0.005 for each pair-wise comparison). Study participants who spent more time watching television, playing video games, or using computers were more likely to have lower 25 (OH) D concentrations (Table 1). Furthermore, participants who ate at a fast food restaurant at least once in the past week were also more likely to have low 25 (OH) D levels.

Table 2 shows mean 25 (OH) D concentrations across BMI categories and other cardiovascular risk factors. Mean 25 (OH) D concentrations were lower among adolescents classified as obese. Although not statistically significant, vitamin D concentrations were lower among those with central obesity. Higher vitamin D concentrations were linked to lower HDL-C. Vitamin D concentrations were also lower among those who had elevated blood pressure and metabolic syndrome but these associations were not significant statistically.

**Table 3:** Multivariate linear regression model for plasma 25 (OH) D concentrations

**Variable**	**β, Estimate ± SE**	**p-value**
Intercept	46.943 ± 12.056	0.000
Age	NS	
Gender	−3.878 ± 1.258	0.002
Fast food per week	−2.103 ± 0.801	0.009
Body mass index	−0.230 ± 0.095	0.017
Physical activity score	1.378 ± 0.698	0.050
HDL cholesterol	NS	
Carbonated soft drink intake	NS	

NS indicates not significant.

Table 3 shows stepwise multivariate linear regression analyses that were conducted to identify independent determinants of Vitamin D concentrations. In this model, all significant (p<0.05) variables from earlier analyses were included to simultaneously adjust for other variables in the model. After adjustment for age, female gender, consumption of fast food per week, and BMI were independently correlated with decreased vitamin D level. Physical activity scores were positively correlated with vitamin D level.

## Discussion

We found a high prevalence of vitamin D deficiency among otherwise healthy adolescents in our school-based study population despite living in a sunny subtropical area (latitude 24º N, longitude 55º E). More importantly a substantially higher proportion of female adolescents had vitamin D deficiency compared to their male counterparts. As part of our exclusion criteria we did not include six subjects who were on treatment for diabetes or hypertension and they might had even lower concentrations of vitamin D. To the best of our knowledge, no population-based data were available from previous studies investigating vitamin D levels in the adolescent population in the UAE.

The prevalence of vitamin D deficiency in adolescents in the UAE is very high, particularly in females, compared to their counterparts in other developed countries where vitamin D fortified foods are available, and people use vitamin D supplements [21,22]. Although the UAE and other Gulf countries have a sunny environment, skin sun exposure is low, and therefore vitamin D deficiency remains one of the major public health problems [23,24].

Both adult and adolescent females observe conservative dress codes that either require covering all body parts including face and hands (Niqab) or all body parts except the face and hands (Hijab). High prevalence of vitamin D deficiency has been noted in adolescent girls who observed conservative dress in Israel and Turkey [25,26]. Studies conducted in a Jordanian population revealed that women who observed Niqab and Hijab had significantly lower concentrations of vitamin D [27].

Inadequate nutritional intake of vitamin D may further contribute to low vitamin D concentrations. Laleye et al. [28] investigated the problem of vitamin D insufficiency in Emirati female students aged 19 to 23 years through a dietary intake assessment and found that over 70% of female students did not consume enough milk and other vitamin-D-rich foods. Data is lacking if UAE milk contains enough vitamin D and studies conducted elsewhere show that fortified milk did not contain the amount of vitamin D claimed on the label [29]. In the study from Al Ain in theUAE, 40% of participants reported consuming fortified milk with a calculated daily average intake of 88 international units of vitamin D [30].

Contrary to existing evidence, our findings did not show a significant association of blood pressure, plasma lipids and metabolic syndrome with 25 (OH) D concentrations. However, our analysis showed an inverse association between 25 (OH) D concentrations and BMI. Even after controlling for age and gender, body mass index remained an independent correlate of 25 (OH) D concentrations, consistent with findings from other studies [29,30]. The sequestration of vitamin D into adipose tissue has been proposed to explain this association [31]. This has important implications for the UAE population as recent figures estimate that 28% of male adolescents and 40% of female adolescents are either overweight or obese [32]. In our study, vitamin D concentrations were positively and significantly (p<0.05) correlated with physical activity and negatively with fast food intake. Other studies have shown that substantially large amounts of carbonated soft drinks were consumed with negligible amounts of milk at fast food places [33].

These findings must be interpreted in light of the acknowledged limitations. We were not able to measure the parathyroid hormone, a measure of bone health loosely associated with vitamin D status [34]. The study was cross-sectional, and therefore, causality cannot be inferred. Information on dietary and physical activity information was obtained by self-reports, with its inherent limitation. We did not evaluate the effect of seasonality as the study was conducted during months of March and April. Given the availability of sun shine almost throughout the year the seasonality may not be an important factor. Our results may not be generalizable to the entire UAE.

## Conclusions

In conclusion, vitamin D deficiency and insufficiency were highly prevalent in females in the United Arab Emirates. Given the potential for serious morbidity, there is a need for urgent monitoring of vitamin D deficiency and timely correction of vitamin D status in the general UAE population by instituting public health measures such as encouragement of moderate sunlight exposure that has been shown to be effective in other Arab population [35].

## Competing interests

The authors declare that they have no competing interests.

## Authors’ contributions

Conceived, designed and implemented the experiments: MAS AEM AK MMN SJM FAM. Conceived, designed, analyzed the data, wrote the paper MAS NN. Wrote the paper AEM AK MMN SJM FAM NN MAS, JAK, ASD. All authors read and approved the final manuscript.

## Pre-publication history

The pre-publication history for this paper can be accessed here:

http://www.biomedcentral.com/1471-2458/13/33/prepub
